# An economic analysis of Chemo Mouthpiece^®^ versus supportive care for the reduction of oral mucositis incidence in patients receiving chemotherapy

**DOI:** 10.57264/cer-2025-0164

**Published:** 2026-02-27

**Authors:** Richard Zuniga, Aidan Dineen, Rosemarie Velasquez, Donghyun D Lee, Anthony Zara, Bonni Tattoli, Megan Bourque, Frank Jacobucci

**Affiliations:** 1 New York Cancer & Blood Specialists, Port Jefferson Station, NY 11776, USA; 2 Value & Evidence, EVERSANA, Victoria, BC V8W2E1, Canada; 3 ChemoMouthpiece, LLC, Closter, NJ 07624, USA

**Keywords:** chemotherapy, cryotherapy, healthcare costs, healthcare resource use, oral mucositis

## Abstract

**Aim:**

Oral mucositis (OM) is a common, burdensome complication of chemotherapy (CT), associated with pain, weight loss and increased infection risk. OM can result in CT dose reductions or delay subsequent cycles, compromising treatment efficacy. Current guidelines recommend oral cryotherapy with basic multiagent oral care for prevention of CT-induced OM. Chemo Mouthpiece^®^ (CMP) is a cryotherapy medical device with FDA 510(k) marketing clearance. A randomized controlled trial demonstrated that CMP effectively reduced the incidence and severity of OM caused by long or short half-life CT regimens in adults, compared with basic supportive oral care (BSOC) alone. The objective of this study was to estimate OM-related clinical and cost impacts associated with reductions in CT-induced OM from use of CMP.

**Materials & methods:**

A model was developed to simulate 1000 adult patients undergoing stomatotoxic CT from a US healthcare payer perspective under two scenarios: optimal practice: 100% using CMP plus best supportive oral care (BSOC); and current practice: 100% using BSOC. Clinical outcomes and costs were modeled to estimate the incremental difference between the two scenarios. Two different time horizons were tested in the model: conservative base case (single CT cycle) and real-world base case (six CT cycles). Additional sensitivity analyses were also conducted.

**Results:**

The cost of CMP in optimal practice was offset by cost savings from other sources, for a net total cost savings. In the conservative base case, optimal practice with CMP use for a single CT cycle was associated with over one million dollars in cost savings compared with current practice ($20,094,565 vs $21,260,470), largely attributed to reduced hospitalization length of stay. Use of CMP in optimal practice was associated with $1166 total cost savings per patient. Of note, substantially greater cost savings were estimated in the real-world base case with a time horizon of six CT cycles (additional savings of $2846 per patient each subsequent CT cycle). Three of the four sensitivity analyses were also found to result in net cost savings.

**Conclusion:**

Adoption of CMP was projected to be associated with reduced OM-associated clinical events and healthcare resource use. For 1000 adults using CMP for one CT cycle, cost savings accumulated to over one million dollars, amounting to $1166 per patient. Results of the real-world base case indicated that the value of CMP is likely underestimated, with cost savings over 15 million dollars, amounting to $15,395 per patient.

## Plain language summary

**What is this article about?:**

This study estimated health outcomes and costs associated with use of Chemo Mouthpiece^®^ (CMP), a novel, reusable, oral cryotherapy device designed to reduce the incidence and severity of oral mucositis (OM) in patients receiving long or short half-life chemotherapy (CT).

**What were the results?:**

Results of the economic model from a US healthcare perspective showed that CMP was associated with lower OM incidence and subsequent cost savings within a single CT cycle, and also when used for six CT cycles, with greater cost savings projected over longer CT cycles.

**What do the results mean?:**

For patients and payers in the US, use of CMP could improve both health and economic outcomes of patients at risk of CT-induced OM. The reusable design of the device allows for greater cost savings over extended CT cycles. Findings from this study could influence the guidelines and healthcare policies on management of CT-induced mucositis.

Oral mucositis (OM) is a common and often debilitating complication of cancer treatment, associated with stomatotoxic chemotherapy (CT) for solid tumors or lymphoma, high-dose CT or radiotherapy (RT) and RT or RT + CT for head and neck cancer [1–7]. These inflammatory and ulcerative lesions of the oral mucosa can cause mild to severe pain, difficulty eating, weight loss and nutritional deficiencies [4,6,8–11]. Patients with OM are also at increased risk for systemic infections, especially those who are immunocompromised or have neutropenia [12]. Further, patient health-related quality of life (HRQoL) is substantially impaired by OM, with mucositis-associated pain severely impacting emotional and physical well-being [6,7,10].

OM typically occurs within 5–14 days of receiving CT [3,13], with the incidence and severity increasing with subsequent CT cycles [14]. For example, over 60% of patients receiving a second cycle of a conventional regimen for breast cancer developed OM compared with 20% in the first cycle [14]. Estimates of the number of patients experiencing OM are uncertain, as the risk of OM varies by cancer types, therapeutic agent and treatment durations. Among the estimated 442,061 patients who receive CT for cancer annually in the US [15], approximately 30% will develop OM [8], equating to ∼132,618 cases of CT-induced OM per year. However, this is likely an underestimation, as a systematic literature review (SLR) reported the incidence of all-grade OM among patients receiving CT for solid tumors ranged from 14.4–81.3% depending on the type of tumor and CT regimen [9], with several reviews reporting OM rates ranging from 20–40% of patients receiving standard CT and 60–85% of patients receiving high-dose CT [3,6,7,13]. Risk factors include smoking, poor oral hygiene, female sex and higher bodyweight [3,6,16].

The clinical and economic burden of OM can be substantial for patients and healthcare systems. OM often necessitates CT dose reductions or treatment cycle delays, which may compromise treatment efficacy [4,6,9]. Unplanned CT dose reductions have been associated with a reduction in 5-year survival from 60% to as low as 10–30% depending on cancer type, with annual mucositis-related deaths largely attributed to CT treatment delays [15]. Patients with OM have longer hospitalizations, greater frequency of analgesic or opioid use and more frequently require medication to manage infections compared with those without OM [8]. Hospitalization costs represent a substantial proportion of total OM-management expenses [17–19], with more severe OM requiring greater healthcare resource use (HCRU) and cost [1,19]. One SLR reported the incremental cost of OM-related hospitalization among patients receiving CT was approximately $3700 per cycle (2017 US dollars [USD]) [20]. Additionally, a cross-sectional study of the 2017 National Inpatient Sample database reported that patients with ulcerative mucositis and healthcare-associated infection had significantly higher total charges than those without infection ($170,569 vs $85,878; 2017 USD) [21]. Indirect costs are also expected to accumulate for patients with OM compared with those without, though additional studies are required.

Despite the burden of OM, prevention and management options remain limited. The 2020 Multinational Association of Supportive Care in Cancer and International Society of Oral Oncology (MASCC/ISOO) guidelines recommend basic multiagent oral care (i.e., best supportive oral care [BSOC]) and oral cryotherapy for patients receiving CT [4]. BSOC emphasizes adherence to routine oral hygiene protocols, such as tooth brushing, flossing, mouthwashes with bland rinses, mucosal hydration and lubrication [4]. Oral cryotherapy induces local hypothermia and vasoconstriction of the mucosal blood vessels, potentially [7,22] limiting mucosal exposure to circulating cytotoxic agents and lower the metabolic rate to reduce inflammation. This may be particularly effective when used during bolus administration of antineoplastic regimens containing agents with short half-lives (e.g., 5-fluorouracil, methotrexate and high-dose melphalan) [7]. An SLR and meta-analysis of 14 studies found that patients treated with oral cryotherapy had a significantly lower risk of developing any grade OM than those without cryotherapy (risk ratio: 0.67; 95% CI: 0.56, 0.81 p < 0.05) [7]. Despite the demonstrated benefits of oral cryotherapy to reduce the incidence and severity of OM, there has been a lack of effective devices available for oncology patients receiving CT.

The Chemo Mouthpiece^®^ (CMP) is an innovative cryotherapy device designed to deliver controlled, localized cooling to the oral mucosa to reduce the incidence and severity of CT-induced OM in a standardized, patient-friendly manner [23,24]. Developed by ChemoMouthpiece, LLC, CMP features a dual-chamber design, including an outer chamber prefilled with a proprietary saline solution and an inner chamber containing filtered water. The single-patient, multiuse device can be reused throughout CT treatment for up to 1 year. CMP has received FDA 510(k) marketing clearance and was granted Breakthrough Device Designation [23,24].

In a pivotal randomized controlled trial (RCT), CMP had favorable outcomes across a representative cohort of patients with various cancer types who receive several common CT regimens, including 5-fluorouracil. Compared with BSOC, CMP use was associated with a significant reduction in the proportion of visits with any patient-reported mucosal pain during the first 14 days of CT cycles 1 and 2 (12.8 vs 23.9%; p < 0.001) [24]. Additionally, patients using CMP had a significantly lower proportion of visits with analgesic use compared with the BSOC only arm (1.8 vs 7.5%; p < 0.001), and a lower incidence of rescue medication use (7.3 vs 3.1%; p = 0.3). No patients in the CMP arm used opioids, compared with 3.3% in the BSOC arm (p < 0.00001, data analyzed by each treatment day that patients reported analgesics use) [25]. These findings are clinically relevant, as opioid use has a substantial impact on patients’ lives, including downstream costs and side effects that can impair clinical outcomes.

The objective of this study was to estimate the OM-related clinical and cost impacts associated with reductions in CT-induced OM from use of CMP, using a predictive health economics simulation model.

## Materials & methods

### Model structure

**Figure 1.**
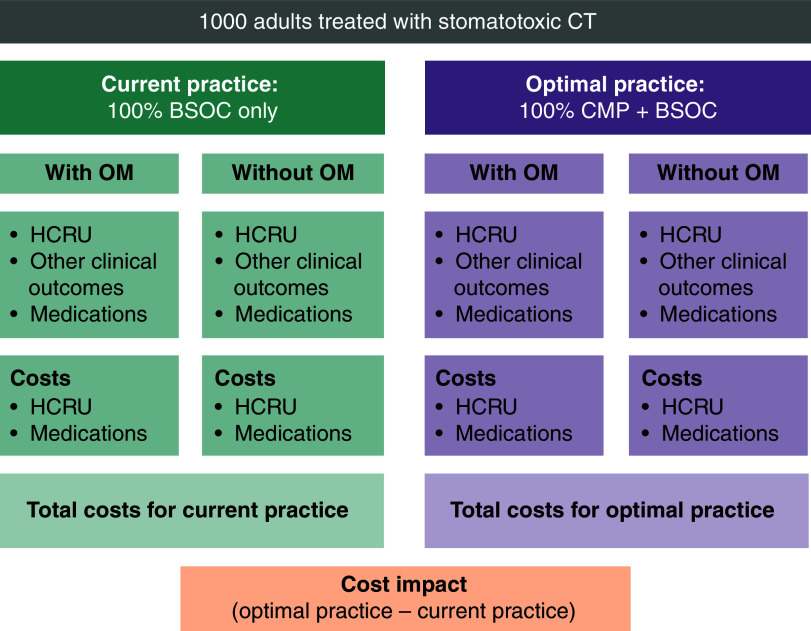
Model structure. BSOC: Best supportive oral care; CMP: Chemo Mouthpiece, CT: Chemotherapy; HCRU: Healthcare resource utilization; OM: Oral mucositis.

This predictive health economics simulation model was developed in Microsoft Excel (Microsoft Corporation, WA, US) and the structure is outlined in Figure 1. A hypothetical cohort of 1000 adult patients undergoing treatment with stomatotoxic CT regimens were compared in two scenarios: optimal practice: 100% using CMP plus BSOC; and current practice: 100% using BSOC only. Patients were stratified by those who did or did not experience OM. The sample size of 1000 patients was chosen for modelling purpose and does not represent a statistically derived sample size. This hypothetical cohort provides results at a clinically meaningful scale and reflects a plausible order of magnitude of cancer patient volumes across US treatment facilities, without implying representativeness of any single institution. Costs for BSOC for patients receiving CT were assumed to be the same in both scenarios and not included. A US healthcare hospital payer perspective was used. Two co-base cases with different time horizons were tested. The conservative base case assumed a single CT cycle even though the CMP device would be re-used for an entire regimen of multiple cycles and the incidence of OM tends to increase with additional cycles. The real-world base case assumed an alternative time horizon of six CT cycles, which aligned with both the average number of CT cycles in the pivotal CMP RCT and the average CT regimen duration observed in real-world practice [24]. This co-base case analysis aimed to understand the cost impacts of CMP use over a more typical duration of a CT regimen (∼six cycles), compared with the conservative base case analysis that captured only a single CT cycle of clinical benefits, despite the full cost of CMP being included. Previous studies have shown that >80% of patients with ovarian cancer [26] and >40% of patients with soft tissue sarcoma [27] received six or more cycles of CT. Further, a real-world cohort study of breast cancer patients implied that 4 cycles of CT is the standard for docetaxel-cyclophosphamide CT regimen [28]. These findings imply that most cancer patients will be treated with multicycle CT regimens, justifying the selection of co-base case analysis assuming multiple CT cycles. Clinical outcomes and costs were modeled to estimate the total incremental costs per patient and cohort between the two scenarios.

### Model parameters

Model inputs are shown in Table 1. Clinical outcomes included incidence of OM, hospitalizations, emergency room (ER) visits, total parenteral nutrition (TPN), medication use, unplanned CT delays or discontinuations, fatigue and weight loss. Costs were not applied to unplanned CT delays or discontinuations, fatigue or weight loss. All costs were in 2024 USD. The CMP device cost was applied as a one-time cost of $2100, and a 20% patient co-pay was assumed. Costs sourced from the literature were inflated to 2024 USD as needed using the Campbell and Cochrane Economics Methods Group - Evidence for Policy and Practice Centre (CCEMG – EPPI-Centre) tool as recommended by Cochrane [29,30].

**Table 1.** Overview of model input parameters and sources.

Parameter (per cycle unless otherwise indicated)				
Clinical inputs by treatment	BSOC	CMP + BSOC	Source(s)	Ref.
Percent of patients with OM	23.9%	12.8%	Zuniga (2025)	[24]
Percent of patients with analgesic use	29.3%	9.2%		
Percent of patients with rescue medication use	7.3%	3.1%	Dembla (2025)	[25]
**Clinical Inputs by OM incidence**	**Patients with OM**	**Patients without OM**	**Source(s)**	
**HCRU**				
Mean general hospital LOS, days	3.9	3.9	Elting (2003)	[8]
Mean additional OM-specific hospital length of stay, days	2.4	0.0		
Mean ER visits, n	1.0	0.8		
Percent of patients requiring TPN	3.0%	0.0%		
**Other clinical outcomes (not costed)**				
Percent of patients with CT dose reduction	25.0%	11.0%	Elting (2003)	[8]
Percent of patients with delayed CT cycle	11.0%	9.0%		
Percent of patients with fatigue	12.0%	5.0%		
Percent of patients with weight loss	61.0%	54.0%		
**Medication**				
Percent of patients requiring antibacterial medication	68.0%	36.0%	Elting (2003)	[8]
Percent of patients requiring antifungal medication	53.0%	12.0%		
Percent of patients requiring antiviral medication	34.0%	6.0%		
Percent of patients using rescue medication on Caphosol	33.3%	33.3%	Assumption	
Percent of patients using rescue medication on MuGard	33.3%	33.3%		
Percent of patients using rescue medication on Gelclair	33.3%	33.3%		
**Unit cost inputs**	**2024 USD**		**Source(s)**	
CMP (one-time cost)	$2100.00		Assumption	
Patient co-pay	20.0%		Assumption	
BSOC	$0.00		Assumption	
General hospital stay, per day^†^	$3207.27		AHA (2022)	[31]
OM-specific hospital stay, per day^‡^	$10,230.64		Satheeshkumar (2022)	[21]
ER visit	$3300.35		Hargraves (2023)	[32]
TPN	$2167.46		Alabama Department of Labor (2024)	[33]
Analgesic pain medication^¶^^,^^#^	$56.88		NAVLIN 2024	[34]
Antibacterial medication^††^	$7.65			
Antifungal medication^‡‡^	$7.28			
Antiviral medication^§§^	$11.31			
Caphosol (mouthwash 1 pack [240 doses, 0.93%])	$712.11			
MuGard (mouthwash 1 240 ml bottle)	$581.94			
Gelclair (oral gel 1 pack, 15 sachets, 100%)	$679.18			

Where applicable, costs were inflated from year reported to 2024 USD using the CCEMG – EPPI-Centre Cost Converter [29,30].

† Median inpatient cost per day, general population.

‡ Mean total cost per visit inflated to 2024 USD ($91,502.66) divided by mean hospital stay (8.9 days).

§ $708.43 per day, conservatively assumed 3.06 days from mild OM population.

¶ Based on representative medications recommended by Stawarz-Janeczek (2020) [35] for treatment of complications secondary to OM.

# Assumed doxepin oral solution (1 bottle, 120 ml [1,200 mg]) sufficient for one cycle of OM-related pain.

†† Assumed cephalexin 1000–4000 mg twice daily for 7–14 days (1 pack, 40 capsules, 500 mg).

‡‡ Assumed fluconazole 200 mg day 1, 100 mg daily for 2 weeks (1 pack, 30 tablets, 100 mg).

§§ Assumed acyclovir 400 mg thrice daily for 7–10 days (1 pack, 100 tablets, 200 mg).

BSOC: Best supportive oral care; CCEMG: Campbell and Cochrane Economics Methods Group; CMP: Chemo Mouthpiece, CT Chemotherapy; EPPI: Evidence for Policy and Practice; ER: Emergency room; HCRU: Healthcare resource utilization; LOS: Length of stay; OM: Oral mucositis; TPN: Total parenteral nutrition; USD: United States Dollar.

A comprehensive targeted literature review completed in July 2024 was used to identify model inputs. Where possible, a single reference was used to inform a given input across treatment arms to reduce bias across different study designs. Studies of populations with CT-induced OM were preferred. The RCT for CMP [24] was used to inform the incidence of OM, analgesic use and rescue medication use for patients in optimal practice versus current practice. A simplifying assumption was made that the efficacy data from the trial collected over two CT cycles represented the percentage of patients who experienced OM in any given CT cycle on each treatment, which supported the choice of a single CT cycle for the time horizon in the conservative base case. Caphosol^®^ (Jazz Pharmaceuticals, Inc., CA, USA), MuGard^®^ (Soleva Pharma LLC, NJ, USA) and Gelclair^®^ (Helsinn Healthcare SA, Lugano, Switzerland) were considered as rescue medications in the present analysis, with an equal distribution for those initiated on these medications (Table 1). The inputs for other clinical outcomes, HCRU and unit costs were reflective of the literature sources and applied per CT cycle. The incidence of clinical events associated with OM and HCRU were informed from a retrospective study of 559 patients who were followed for development of OM and gastrointestinal mucositis [8]. To estimate hospitalization length of stay (LOS), it was assumed that patients could experience hospitalization regardless of OM status, and therefore the model applied LOS to all patients. Given potential uncertainties associated with this model input, a conservative approach was taken to estimate the incremental burden of OM by applying a general hospitalization cost per day for the LOS that was common for patients with and without OM, with an OM-specific hospitalization cost applied only to the additional LOS period reported for patients with OM.

Key model results included the reduction in OM incidence between the optimal [ractice and current practice scenarios and the difference in total costs between scenarios (per patient and total cohort). Differences in HCRU and other clinical events were also captured.

### Sensitivity analyses

Several sensitivity analyses were conducted to explore alternative inputs or scenarios, including: CMP plus BSOC versus ice chips (IC) plus BSOC; alternative OM-specific hospitalization costs; alternative OM-specific hospitalization LOS; and exclusion of rescue medication use. Relevant parameters and assumptions for the sensitivity analyses are summarized in Table 2.

**Table 2.** Overview of model input parameters and sources for all analyses.

Analysis/parameters	Base case value(s)	Sensitivity analysis value(s)	Source(s)	Refs.
**Co-base case analyses**				
Cycles, n	1* and 6**	N/A	*Conservative assumption **Assumption based on typical chemo regimen duration ranges^†^	[36–41]
**Sensitivity analysis 1: CMP + BSOC vs IC + BSOC**				
Market distribution by scenario	Current practice: 100.0% BSOC Optimal practice: 100.0% CMP + BSOC	Current practice: 57.0% BSOC 43.0% IC + BSOC Optimal practice: 64.5% CMP + BSOC 10.9% BSOC 24.6% IC + BSOC	Assumption, market research data^‡^	
Percent of patients with OM	IC + BSOC: not applicable	IC + BSOC: 16.6%	Sorensen (2008); assumption^§^	[42]
Percent of patients with analgesic use		IC + BSOC: 20.3%		
Percent of patients with rescue medication use		IC + BSOC: 5.1%		
**Sensitivity analysis 2: alternative OM-specific hospitalization costs**				
OM-specific hospital stay cost, per day	$10,230.64	$3207.27	Assumption: same as general hospital stay	
**Sensitivity analysis 3: alternative OM-specific hospitalization LOS**				
Mean general hospital LOS, days	With OM: 3.9 Without OM: 3.9	With OM: 0.0 Without OM: 3.9	Assumption: entire LOS with OM has OM-specific hospitalization cost	
Mean additional OM-specific hospital length of stay, days	With OM: 2.4 Without OM: 0.0	With OM: 6.3 Without OM: 0.0		
**Sensitivity analysis 4: no rescue medication costs**				
Costs for rescue medication	Included	Excluded	Assumption	

All four sensitivity analyses were performed on each of the co-base case analysis. Results of the sensitivity analyses based on real-world base case is presented in the Supplementary Materials.

† Breast cancer 3–12 cycles (doxorubicin plus cyclophosphamide [AC] with or without paclitaxel [T], docetaxel-based regimens) [36,37]; colorectal cancer 6–12 cycles (leucovorin calcium plus fluorouracil plus irinotecan hydrochloride [FOLFIRI] with or without bevacizumab) [38,39]; lymphoma 4–8 cycles (AC) [40,41].

‡ Market share projections based on internal market research. [43].

§ Comparative efficacy of IC plus BSOC was estimated based on a naive indirect comparison using data from Sorensen [42] (e.g., relative risk [RR] of OM = 0.6939 [p = 0.0163; for IC plus BSOC vs BSOC alone]; RR applied to the risk of OM for patients on BSOC only from Zuniga [24], the estimated risk of OM for IC plus BSOC was 16.6%).

BSOC: Best supportive oral care; CMP: Chemo Mouthpiece, IC: Ice chips; LOS: Length of stay; OM: Oral mucositis.

## Results

The economic and clinical results of the co-base case analyses are summarized in Table 3 & Supplementary Table 1, respectively. For 1000 adult patients receiving stomatotoxic CT, 111 fewer patients would be predicted to experience OM in the optimal practice versus current practice scenarios (128 vs 239). Compared with current practice, the optimal practice scenario was also associated with reductions in HCRU (hospital LOS, ER visits and TPN use), lower incidence of other undesirable clinical outcomes (CT dose reduction or delay, fatigue and weight loss), and decreased medication use (opioid analgesic, rescue, antibacterial, antifungal and antiviral).

**Table 3.** Summary of economic results for conservative and real-world base case analyses, per 1000-patient cohort.

Economic outcome	Current practice	Optimal practice	Incremental
CMP	$0.00	$2,100,000.00	$2,100,000.00
Patient co-pay	$0.00	$420,000.00	$420,000.00
Net CMP cost for payer	$0.00	$1,680,000.00	$1,680,000.00
**Conservative base case**			
HCRU			
Hospitalization	$18,376,648.10	$15,651,205.61	-$2,725,442.50
ER visit	$2,798,036.73	$2,724,768.96	-$73,267.77
TPN	$15,540.68	$8323.04	-$7217.64
Subtotal	$21,190,225.52	$18,384,297.61	-$2,805,927.91
Medication			
Analgesic medication	$16,647.80	$5250.46	-$11,397.34
Rescue medication	$48,015.26	$20,390.04	-$27,625.22
Antibacterial medication	$3339.07	$3067.34	-$271.73
Antifungal medication	$1586.97	$1255.65	-$331.31
Antiviral medication	$1435.47	$1083.95	-$351.51
Subtotal	$71,024.57	$31,047.44	-$39,977.11
Total	$21,261,250.09	$20,094,565.06	-$1,165,905.52
**Real-world base case**			
HCRU			
Hospitalization	$110,255,208.62	$93,902,553.65	-$16,352,654.98
ER visit	$16,788,220.38	$16,348,613.76	-$439,606.62
TPN	$93,244.09	$49,938.26	-$43,305.83
Subtotal	$127,136,673.10	$110,301,105.67	-$16,835,567.43
Medication			
Analgesic medication	$99,886.83	$31,502.77	-$68,384.06
Rescue medication	$288,091.58	$122,340.26	-$165,751.32
Antibacterial medication	$20,034.43	$18,404.06	-$1630.37
Antifungal medication	$9521.80	$7533.93	-$1987.88
Antiviral medication	$8612.79	$6503.70	-$2109.09
Subtotal	$426,147.44	$186,284.72	-$239,862.71
Total	$127,562,820.53	$112,167,390.39	-$15,395,430.14

CT: Chemotherapy; CMP: Chemo Mouthpiece^®^, ER: Emergency room; HCRU: Healthcare resource utilization; OM: Oral mucositis; TPN: Total parenteral nutrition.

The use of CMP in the optimal practice scenario was associated with an estimated upfront cost of $2100 per patient. The conservative base case assumed benefits from reduction in HCRU over a single CT cycle, while the real-world base case explored the estimated CMP cost and benefits over six CT cycles. Assessing the cohort-level impact in the conservative base case, optimal practice with CMP use for a single cycle of CT was associated with over one million dollars in cost savings compared with current practice ($20,094,565 vs $21,260,470), which was substantially greater in the real-world base case showing over 15 million dollars ($127,562,820 vs $112,167,390) in cost savings. In both analyses, cost offsets were primarily attributed to reductions in HCRU: hospitalization LOS (95.8%); ER visits (2.6%) and TPN (0.3%). Reductions in medication use contributed to 1.4% of the observed total cost offsets. Assessing the patient-level impact in the conservative base case, use of CMP in Optimal Practice was associated with at least $1166 per patient in total cost savings compared with current practice, with $2806 HCRU and $40 in medication cost offsets, respectively. Additional cost savings of $2846 per additional CT cycle were estimated based on savings from HCRU. In the real-world base case analysis, substantially greater per-patient cost savings were observed ($15,395 per patient vs $1166 per patient).

**Figure 2.**
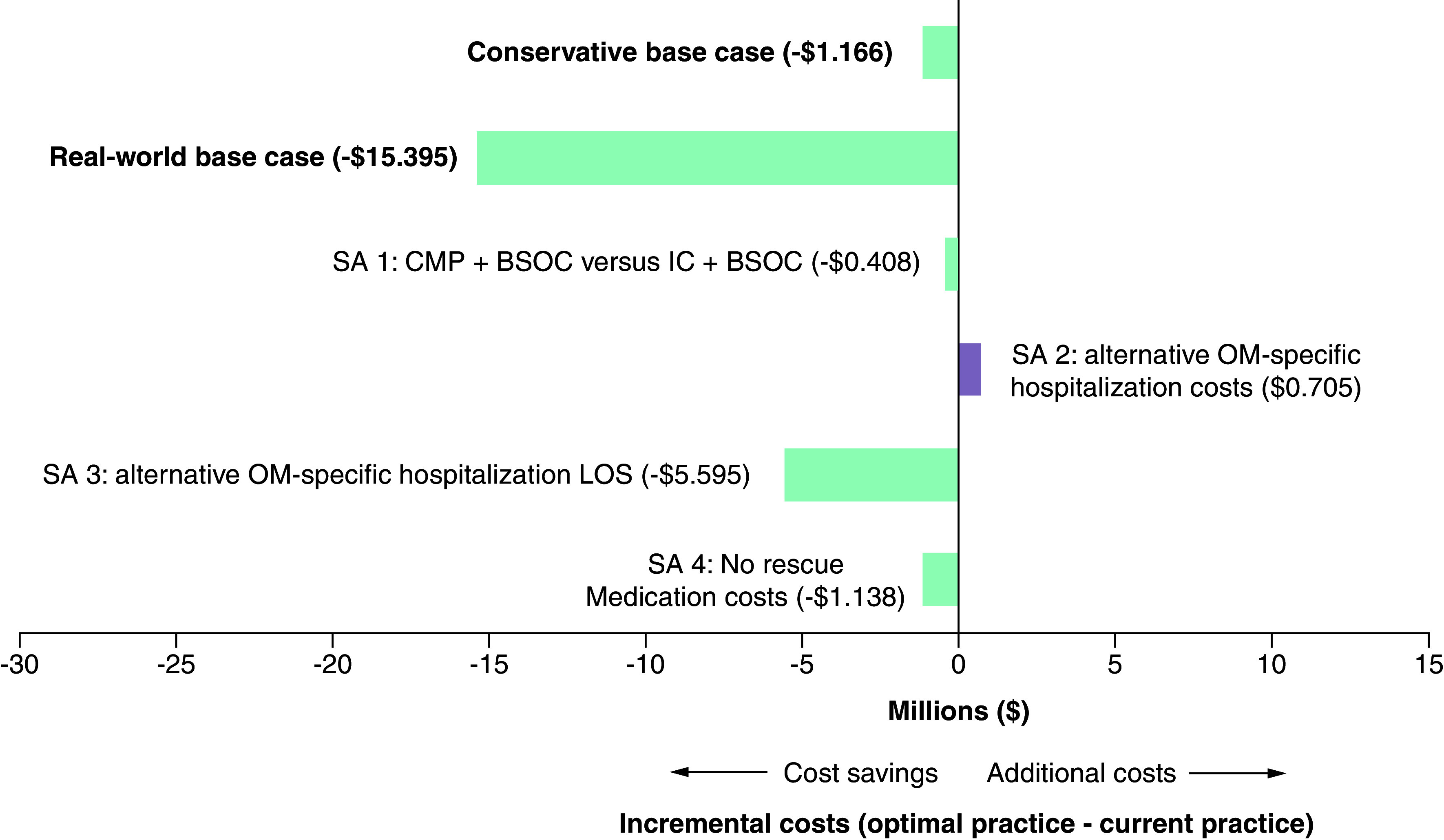
Incremental economic results of sensitivity analyses based on the conservative base case, per 1000-patient cohort. BSOC: Best supportive oral care; CMP: Chemo Mouthpiece^®^; IC: Ice chips; LOS: Length of stay; SA: Sensitivity analysis.

Three of the four sensitivity analyses performed on the conservative base case were also found to result in net cost savings from the hospital payer perspective (Figure 2). The analysis exploring a more complex current practice and optimal practice treatment landscape with some patients on IC plus BSOC (with an estimated reduction in OM compared with BSOC alone) was also found to be cost saving ($407,974). The sensitivity analysis assuming the same OM and non-OM cost per hospitalized day was found to have incremental costs ($705,174), with an incremental cost per patient of $705 for those treated with CMP. Conversely, the sensitivity analysis assuming patients with OM will incur the OM-specific hospitalization cost for the entire LOS was found to greatly increase the cost savings relative to the base case ($5,594,749). Removing rescue medication costs had a minimal impact on results ($1,138,280) compared with the conservative base case. On the other hand, all four sensitivity analyses performed on the real-world base case showed net cost savings from the hospital payer perspective (Supplementary Figure 1).

## Discussion

Stomatotoxic CT-induced OM is an often overlooked but potentially serious clinical event that many patients unfortunately experience. The risk of OM increases with each cycle of CT [14], with increased risk of lengthy hospitalization, ER visits, and use of analgesics among other medications [8]. There are few recommended treatments to reduce the incidence of CT-induced OM, with much of current clinical practice focused on the management of OM after it occurs [4,6]. The CMP device has been shown in an RCT to reduce the incidence of OM induced by long or short half-life CT regimens, in addition to the use of analgesic medication [24]. This analysis explored the clinical and cost outcomes associated with reduction in CT-induced OM, from a hospital payer perspective.

The reduction in the incidence of CT-induced OM associated with use of CMP was found to drive potential reductions in HCRU, other clinical events and medication use. When considered across a cohort of 1000 patients for a single CT cycle these reductions in many cases could be extensive: 266 fewer days in hospital; 200 fewer patients with analgesic use; and 112 patients with less medication use for infections. Importantly, reductions in other unfavorable OM-related clinical events could also be possible: 17 fewer patients with CT dose reduction or delayed cycle and 14 fewer patients with fatigue or weight loss.

The conservative base case analysis where all patients were assumed to be on CMP plus BSOC compared with BSOC alone demonstrated substantial cost savings of over one million USD for the hypothetical cohort of 1000 patients. The primary driver of cost savings was avoidance of additional LOS associated with OM hospitalizations, with reductions in ER visits and medication use also contributing to cost offsets. This is a noteworthy finding, especially considering the time horizon was a single cycle. The cost of the CMP device will be further offset by subsequent cycles where the risk of CT-induced OM increases. This conclusion is supported by the findings from the real-world base case, where time horizon of six CT cycles resulted in $15,395 in cost savings per patient. Cost savings were also observed in the sensitivity analysis 1 that considered some patients on IC plus BSOC in current practice, whereas the most conservative sensitivity analysis 2 (assuming no added cost for OM-specific hospitalizations) resulted in a cost offset of 54% of the total CMP cost per patient. Conversely, sensitivity analysis 3, assuming a more realistic cost of OM-related hospitalization from real-world evidence for the entire LOS [21], was found to increase cost savings compared with the base case (refer to Supplementary Figure 2).

This study had several strengths. The clinical efficacy estimates were based on robust RCT-based efficacy data for CMP. Estimates for OM-related HCRU were from a single comprehensive observational study comparing resource use between populations with and without OM, which avoided potential bias from a mix of study sources informing model inputs. Numerous sensitivity analyses exploring alternative inputs and assumptions were performed that generally supported the base case conclusion of cost savings, and suggested the base case may have been conservative. For example, OM-specific hospitalization costs were conservatively only applied to additional LOS beyond that for patients without OM, the cost of which may be quite large [20].

The study also had limitations. As described, time horizon in the conservative base case was set to one CT cycle, which underestimated the full cost-offsets associated with a multicycle CT regimen, as the CMP device can be reused for up to 1 year. Although this has limited potential impacts of the uncertainties regarding the long-term efficacy of CMP beyond the two cycles reported in the RCT, data from the literature has informed that the risk of OM and cumulative damaging increases with each CT cycle, implying and highlighting the benefits of early CMP use. Results of the real-world base case analysis confirmed the underestimated benefit from the conservative base case analysis; however, given the uncertainties and assumptions used in the current model-based study, the results should be interpreted with caution. Another limitation was that heterogeneity throughout the literature in OM incidence and resource use required simplifying assumptions to facilitate modelling. It is known that there is large variability in OM risk by cancer type, chemotherapy regimen, number of CT cycles and patient factors [3,6,9,16]. However, in absence of clinical efficacy data, subgroup-specific analyses could not be performed. An area for future research that was not explored here would be to compare the relative economic outcomes for different cancer types and/or regimens.

Although oral cryotherapy is recommended by clinical practice guidelines, use of available methods of administration (i.e., ice chips) may be limited due to adverse events including numbness, nausea and tooth hypersensitivity and pain [44,45]. Despite limited available data to inform comparisons between CMP and IC for patients receiving CT, a naive comparison was performed that did not consider differences in study design and patient characteristics. Using the best currently available data, cost benefits of CMP versus IC were estimated. However, this likely underestimate benefits of CMP in other facets such as improved CT regimen adherence, patient quality of life and fewer healthcare provider resources needed for cryotherapy administration.

## Conclusion

This study provides an overview of the clinical and economic benefits of shifting current clinical practice to use of CMP for the reduction of OM associated with adults receiving stomatotoxic CT. Adoption of CMP would be anticipated to lead to reductions in OM-associated clinical events, which could improve patient quality of life and reduce resource use. The conservative base case showed cost savings of $1166 per patient accumulated to over one million dollars in downstream net cost savings, and the real-world base case demonstrated substantially greater cost savings of $15,385 per patient adding up to over 15 million dollars. Sensitivity analyses demonstrated that the base case was robust to alternative inputs and assumptions. Further, the model did not account for the added value of reductions to interruptions of CT regimens, improvements to quality of life and reductions to dependency on pain medications.

## Summary points

- Oral mucositis (OM) is a common complication of chemotherapy (CT) associated with pain, weight loss and increased infection risk.
- OM can also lead to CT dose reductions or treatment delays, which can compromise clinical outcomes.
- This study utilized an economic model to simulate a hypothetical cohort of 1000 adult patients undergoing stomatotoxic CT from a US healthcare payer perspective under two scenarios: optimal practice: 100% using Chemo Mouthpiece^®^ (CMP) plus best supportive oral care (BSOC); and current practice: 100% using BSOC.
- In the economic model, the cost of CMP in optimal practice was offset by cost savings from other sources, for a net total cost savings.
- For the 1000 patient cohort, optimal practice with CMP use for a single CT cycle was associated with over one million dollars in cost savings compared with current practice. Use of CMP in optimal practice was associated with $1166 total cost savings per patient.
- Substantially greater cost savings ($15,395 per patient) were estimated in the real-world base case with a time horizon of six CT cycles, which resemble CT cycle duration in real-world practice.
- Overall, findings of this study suggest that adoption of CMP is associated with reduced OM-associated clinical events and healthcare resource use.

## Supplementary Material


