# Supplementary material for: An economic analysis of Chemo Mouthpiece® versus supportive care for the reduction of oral mucositis incidence in patients receiving chemotherapy
Source: J Comp Eff Res. 2026 Feb 27;15(3):e250164. doi: 10.57264/cer-2025-0164 (PMC12976640; doi:10.57264/cer-2025-0164)
Supplement: Supplementary file 1 [file cer-15-250164-s1.docx]

**Supplementary Material to the article:** An Economic Analysis of Chemo Mouthpiece^®^ versus Supportive Care for the Reduction of Oral Mucositis Incidence in Patients Receiving Chemotherapy.

**AUTHORS:** Richard Zuniga^1^, Aidan Dineen^2^, Rosemarie Velasquez^1^, Donghyun D. Lee^2^, Anthony Zara^2^, Bonni Tattoli^3^, Megan Bourque^2^, Frank Jacobucci^3^

**AFFILIATIONS:** 1. New York Cancer and Blood Specialists, Port Jefferson Station, NY, USA, 2. Value and Evidence, EVERSANA, Burlington, ON, Canada, 3. ChemoMouthpiece, LLC, Closter, NJ, USA.

**CORRESPONDING AUTHOR:** Richard Zuniga; rzuniga@nycancer.com.

**Supplemental Fig. 1 Incremental economic results of sensitivity analyses based on Real-world base case, per 1,000-patient cohort.**

**
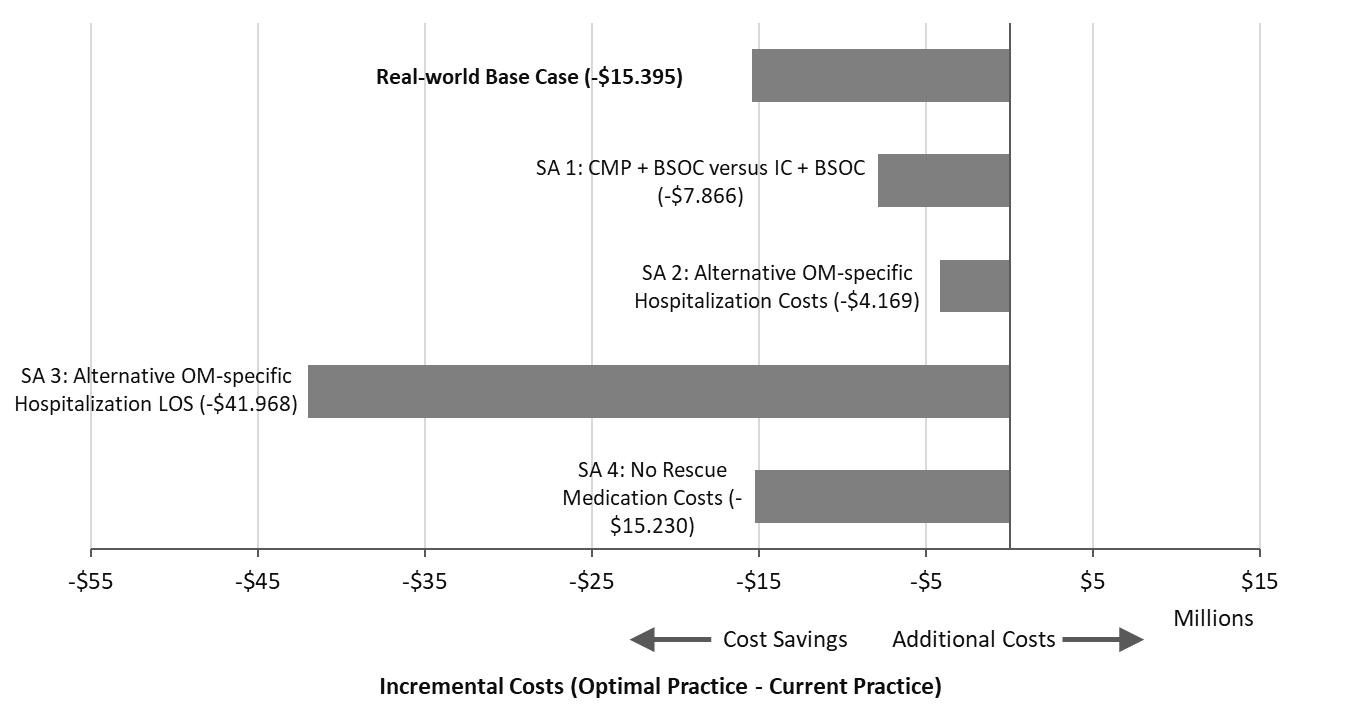
**

Abbreviations: BSOC = best supportive oral care; CMP = Chemo Mouthpiece^®^; IC = ice chips; LOS = length of stay.

**Supplemental Fig. 2** **Assumptions used in sensitivity analyses 2 and 3**


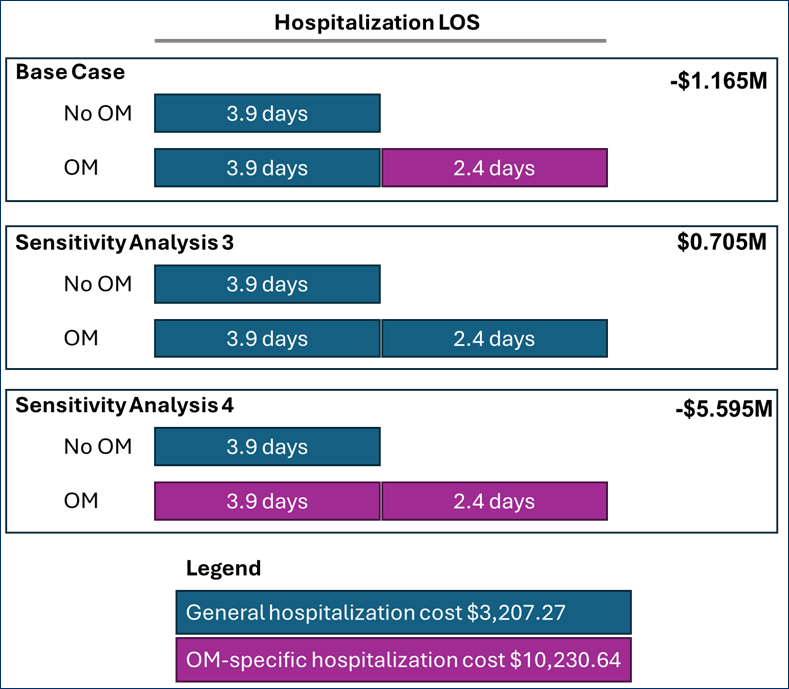


Note: In the base case analysis, OM-specific hospitalization cost ($10,230) was applied to the additional 2.4 days associated with OM hospitalization (not the full LOS of 6.3 days), which was a conservative approach. Sensitivity analysis 2 was the most conservative assumption and explored the results when the cost of hospitalization per day is the same general cost ($3,207) for those experiencing OM (6.3 days LOS) vs. no OM (3.9 days LOS). Sensitivity analysis 3 explored the results are when the cost of hospitalization per day is the full OM-specific hospitalization cost ($10,230, 6.3 days LOS) vs. general hospitalization cost for no OM ($3,207, 3.9 days LOS). Abbreviations: LOS = length of stay; OM = oral mucositis.

**Supplemental Table 1. Summary of clinical results for co-base cases per 1,000-patient cohort**

| **Clinical Outcome** | **Current Practice** | **Optimal Practice** | **Incremental** |
| --- | --- | --- | --- |
| Number of patients with OM | 239.00 | 128.00 | -111.00 |
| ***Conservative base case*** | | | |
| ***HCRU*** | | | |
| Total hospital LOS, days | 4,473.60 | 4,207.20 | -266.40 |
| Total ER visits | 847.80 | 825.60 | -22.20 |
| Number of patients with TPN use | 7.17 | 3.84 | -3.33 |
| ***Other Clinical Outcomes (not costed)*** | | | |
| Number of patients with CT dose reduction | 143.46 | 127.92 | -15.54 |
| Number of patients with delayed CT cycle | 94.78 | 92.56 | -2.22 |
| Number of patients with fatigue | 66.73 | 58.96 | -7.77 |
| Number of patients with weight loss | 556.73 | 548.96 | -7.77 |
| ***Medication*** | | | |
| Number of patients with analgesic use | 292.68 | 92.31 | -200.38 |
| Number of patients with rescue medication use | 73.00 | 31.00 | -42.00 |
| Number of patients with antibacterial use | 436.48 | 400.96 | -35.52 |
| Number of patients with antifungal use | 217.99 | 172.48 | -45.51 |
| Number of patients with antiviral use | 126.92 | 95.84 | -31.08 |
| ***Real-world base case*** | | | |
| ***HCRU*** | | | |
| Total hospital LOS, days | 26,841.60 | 25,243.20 | -1,598.40 |
| Total ER visits | 5,086.80 | 4,953.60 | -133.20 |
| Number of patients with TPN use | 43.02 | 23.04 | -19.98 |
| ***Other Clinical Outcomes (not costed)*** | | | |
| Number of patients with CT dose reduction | 860.76 | 767.52 | -93.24 |
| Number of patients with delayed CT cycle | 568.68 | 555.36 | -13.32 |
| Number of patients with fatigue | 400.38 | 353.76 | -46,62 |
| Number of patients with weight loss | 3,340.38 | 3,293.76 | -46.62 |
| ***Medication*** | | | |
| Number of patients with analgesic use | 1,756.10 | 553.85 | -1,202.25 |
| Number of patients with rescue medication use | 438.00 | 186.00 | -252.00 |
| Number of patients with antibacterial use | 2,618.88 | 2,405.76 | -213.12 |
| Number of patients with antifungal use | 1,307.94 | 1,034.88 | -273.06 |
| Number of patients with antiviral use | 761.52 | 575.04 | -186.48 |

Abbreviations: CT = chemotherapy; CMP = Chemo Mouthpiece^®^; ER = emergency room; HCRU = healthcare resource utilization; LOS = length; OM = oral mucositis; TPN = total parenteral nutrition.
